# A First-Out Alarm Detection Method via Association Rule Mining and Correlation Analysis

**DOI:** 10.3390/e26010030

**Published:** 2023-12-27

**Authors:** Ding Li, Xin Cheng

**Affiliations:** 1 School of Electrical and Information Engineering, Wuhan Institute of Technology, Wuhan 430205, China; 2 School of Automation, China University of Geosciences, Wuhan 430074, China; xin-cheng@cug.edu.cn

**Keywords:** alarm system, alarm correlation, first-out alarms, association rule, correlation analysis

## Abstract

Alarm systems are commonly deployed in complex industries to monitor the operation status of the production process in real time. Actual alarm systems generally have alarm overloading problems. One of the major factors leading to excessive alarms is the presence of many correlated or redundant alarms. Analyzing alarm correlations will not only be beneficial to the detection of and reduction in redundant alarm configurations, but also help to track the propagation of abnormalities among alarm variables. As a special problem in correlated alarm detection, the research on first-out alarm detection is very scarce. A first-out alarm is known as the first alarm that occurs in a series of alarms. Detection of first-out alarms aims at identifying the first alarm occurrence from a large number of alarms, thus ignoring the subsequent correlated alarms to effectively reduce the number of alarms and prevent alarm overloading. Accordingly, this paper proposes a new first-out alarm detection method based on association rule mining and correlation analysis. The contributions lie in the following aspects: (1) An association rule mining approach is presented to extract alarm association rules from historical sequences based on the FP-Growth algorithm and J-Measure; (2) a first-out alarm determination strategy is proposed to determine the first-out alarms and subsequent alarms through correlation analysis in the form of a hypothesis test on conditional probability; and (3) first-out rule screening criteria are proposed to judge whether the rules are redundant or not and then consolidated results of first-out rules are obtained. The effectiveness of the proposed method is tested based on the alarm data generated by a public simulation platform.

## 1. Introduction

Modern industrial systems are developing towards automation, integration, and intelligence, and the scales of industrial processes are also expanding. At the same time, this also imposes high requirements for the safety, stability, and efficiency of the operation of production processes. Alarm systems are the core components of modern industrial facilities, and are used for real-time monitoring of the operational status of all aspects of production. They generate alarms to notify operators of abnormal situations and assist operators to take timely measures to ensure safe operation. Actual alarm systems generally have the alarm overloading problem. Massive alarm messages in real-time operation not only increase the workload of operators, but also make them ignore key alarms, which can easily lead to catastrophic accidents [1,2].

One of the major factors leading to excessive alarms is the presence of correlated or redundant alarms. An actual industrial process generally contains a large number of monitored variables; abnormalities generated in one place continuously propagate through interconnected equipment, causing a series of correlated alarms to appear in a certain order [2,3]. Analyzing alarm correlations will not only be beneficial to the detection of and reduction in redundant alarm configurations, but also help to track the propagation of abnormalities among alarm variables.

In the literature, there exist a variety of correlation analysis methods to detect correlated alarms and measure the correlation levels. References [4,5] studied and compared quite a few similarity coefficients and correlation measurements for detection of correlated alarms; in [5], Sorgenfrei and Jaccard coefficients were selected to measure the correlations between binary-valued alarm signals. An event correlation analysis method was designed in [6] to identify the correlated alarms, as well as to detect the relations between alarms and actions. In view of the negative influences caused by random delays between alarm occurrences, binary signals were transformed into continuous-valued pseudo signals in [7,8], and reference [8] proposed a statistical test based on the random occurrence delays to determine correlated alarms. References [9,10] divided a whole alarm sequence into several blocks, and further measured the correlation of the whole sequence by detecting the correlations of each block. A general weight-based multi-state sequential algorithm for correlation analysis was proposed to measure the alarm correlation among different tags in [11].

Correlations do not imply directions of influences between variables. Accordingly, causality inference, association rule mining, and sequential pattern mining have been exploited to find such directions when detecting correlated alarms. Among these approaches, causality inference detects the causal relations from historical data complemented by process knowledge [12]; commonly used methods include Transfer Entropy [13,14,15], Granger causality [16], and qualitative trend analysis [17]. In [14,15], transfer entropies were exploited and modified to detect the causal relations between alarm signals. An active dynamic transfer entropy was proposed in [18] and a K2-algorithm-based transfer entropy approach was proposed in [19] to conduct alarm causality analysis. In addition, data mining approaches are effective in extraction of interesting association rules and sequential patterns, and have been applied to find temporal dependencies between alarms from historical alarm and event data [20,21,22,23,24]. For instance, reference [23] detected association rules of mode-dependent alarms by examining the relations between alarms and mode events; in [24], a pattern extraction method was proposed to detect alarm sequential patterns from historical alarm floods.

According to the above literature survey, existing alarm correlation analysis methods identify correlated alarms and redundant alarms, which can help to rationally configure alarms, and thus eliminate invalid alarms. The causality inference and data mining approaches find directed relations between alarms, and are helpful to track the propagation of abnormalities. As a special problem in correlated alarm detection, the research on first-out alarm detection is very scarce. A first-out alarm is known as the first alarm that occurs in a series of alarms [25]. Detection of first-out alarms aims at identifying the first alarm occurrence from a large number of alarms, thus ignoring the subsequent correlated alarms to effectively reduce the number of alarms and prevent alarm overloading. How to identify first-out alarms from the alarm sequences, eliminate subsequent redundant alarms, and improve the decision support for the operators is an urgent problem. For the first time, a previous study [26] proposed a first-out alarm detection method that detects the correlation of different alarms and the temporal sequential relationship to find the first occurrence of alarms. However, the method is based on paired binary signals and is computationally intensive, and is concerned only with the long-term dependency, so would therefore miss many rules of short-term relations.

Based on the above problem analysis, this paper proposes a new first-out alarm detection method based on association rule mining and correlation analysis, which discovers first-out alarms in alarm sequences from historical alarm and event data using pattern mining, first-out alarm determination, and first-out rule screening. The contributions lie in the following aspects: (1) an association rule mining approach is presented to extract alarm association rules from historical sequences based on the FP-Growth algorithm and J-Measure; (2) a first-out alarm determination strategy is proposed to determine the first-out alarms and subsequent alarms through correlation analysis in the form of a hypothesis test on conditional probability; and (3) first-out rule screening criteria are proposed to judge whether the rules are redundant, and then consolidated results of first-out rules are finally obtained. The effectiveness of the proposed method is tested based on the alarm data generated by a public chemical simulation platform.

The rest of the paper is organized as follows: Section 2 introduces the industrial alarm system and alarm data, and describes the first-out alarm detection problem. Section 3 proposes the systematic method for first-out alarm detection. Section 4 presents a case study to illustrate the method, and the conclusions are drawn in the last section.

## 2. Problem Description

This section introduces the basics of industrial alarm systems and alarm data, and leads to the problem of first-out alarm detection.

### 2.1. Preliminaries of Industrial Alarm System

**Figure 1:**
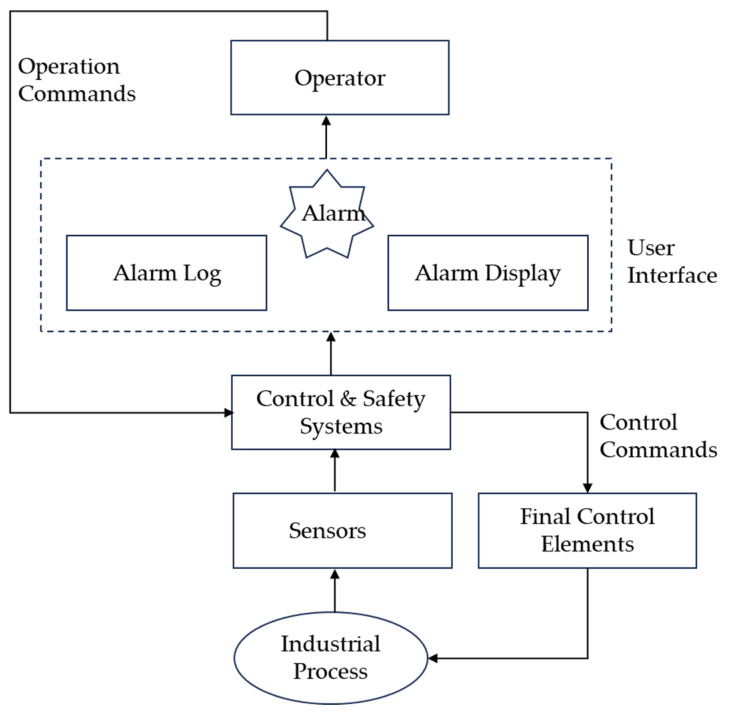
Schematic and functionality of an industrial alarm system.

Industrial alarm system consists of control and safety systems and user interfaces. As shown in Figure 1, when the value of the monitored variable exceeds the normal value range, the corresponding alarm signal changes from “0” to “1”, and the system will send out the alarm prompts to the operator via visible or audible methods; then, the plant operator takes actions to respond to alarms and seeks strategies to fix the issues. When the operator takes the appropriate measures, the operating instructions are sent to the controller, which controls the actuator to make the corresponding executive action, so that the values of the abnormal variables are restored to the normal ranges to ensure that the production is restored to a safe state. Good performance of an alarm system is of great significance to ensure industrial safety, high efficiency, and stable production.

Modern industrial systems have complex equipment interconnections and topologies, and failures occurring in one part may propagate through the system, causing other nodes to also fail, thus generating a large number of associated alarms. Too many alarms would degrade the performance of the alarm system and increase the operator’s workload. On one hand, a large number of alarms are not only detrimental to the operator’s ability to handle the correct alarms, but also make the operator ignore the correct alarms due to the “crying wolf” effect, resulting in the alarm system being useless; on the other hand, when there is a flood of alarms, even if all of the alarms are correct, due to the excessive number of alarms, it would go beyond the ability of operators to deal with them. Therefore, eliminating nuisance alarms in an alarm system is of great significance for the improvement of alarm monitoring performance.

An essential aspect of industrial alarm systems is to minimize false alarms and ensure prompt abnormality detection. Such objectives must be considered in the design of industrial alarm systems via techniques such as alarm limit optimization [27], alarm filters [28], delay timers [29], and deadbands [30], which are usually applied to continuous-valued process data to balance the false positive rate (also known as the false alarm rate) and false negative rate (also known as the missed alarm rate). It needs to be clarified that the first-out alarm detection in this work has a different objective, as it is a different task from alarm system design and it relies on alarm and event data rather than continuous-valued process data. Detection of first-out alarms aims to detect the relations between alarms, and then extract rules of first-out alarms, so as to reduce redundant information during alarm monitoring. Thus, it does not need to consider the minimization of the false positive rate (or false alarm rate) and the false negative rate (or missed alarm rate). In this work, the proposed method is specifically designed for detection of first-out alarms. The results can help to reduce the number of alarms and prevent alarm overloading by ignoring subsequent alarms occurring after the first-out alarms.

### 2.2. Introduction of Industrial Alarm Data

In an alarm system, high and low alarm thresholds are usually configured, so that a binary alarm signal is generated by comparing the continuous process signal with the thresholds. For a continuous process variable x, denote the corresponding high and low alarm thresholds as $x_{HAL}$ and $x_{LA}$, respectively. When the measured signal x(t) exceeds the alarm thresholds, an alarm occurs and is recorded as “1”; otherwise, it is in the normal state and is recorded as “0”. Thus, a binary-valued alarm signal is generated by

(1) $$a\left(t\right)=\left\{\begin{array}{c} 1\;\;x\left(t\right)\geq x_{HA}\;\mathrm{or}\;x\left(t\right)\leq x_{LA},\\ 0\;\mathrm{otherwise}. \end{array}\right.$$

**Figure 2:**
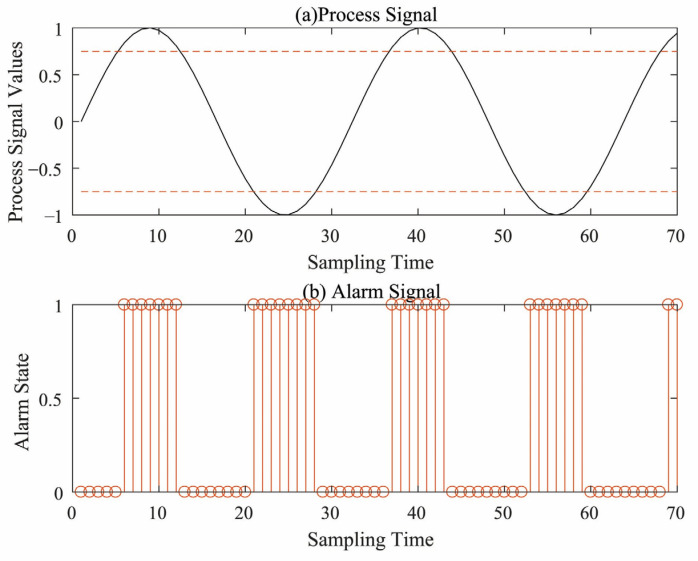
An example of alarm signal generation.

The binary-valued alarm signal is a time series consisting of “0” and “1”, reflecting the state of a single measurement variable at the sampling point. An example of the binary valued alarm signal obtained from the process signal is shown in Figure 2.

With deployment of the decentralized control systems and programmable logic controllers, the configuration of alarms and historization of alarm data have become easy. Usually, the historical alarm information is stored in the alarm logs in the form of alarm sequences. An alarm sequence is a sequence of time-stamped alarms ordered chronologically, and is denoted by

(2) $$F_{k}=<a_{1},a_{2},\cdots ,a_{n}>$$

where $F_{k}$ indicates the *k*th alarm sequence; *n* denotes the number of alarm events within this sequence; and $a_{i}$ denotes the *i*th alarm event. Table 1 shows an example of alarm and event data. In this table, alarm and event data contain alarm tags, timestamps, priorities, and other information. The alarm tags in the alarm dataset are extracted, and the alarm sequences can be obtained by arranging them in the order of occurrence time.

### 2.3. First-Out Alarm Detection Problem

In modern industrial processes, where different devices are physically connected, an abnormality in one unit is likely to be transmitted to other devices through the material, energy, and information flows, resulting in a large number of correlated alarms. Alarm correlation analysis enables operators to focus on the most important alarms in a series of alarms, thus ignoring invalid alarms, preventing alarm overload, and effectively improving the performance of the alarm system. A large number of data-driven alarm correlation analysis methods have been proposed in the existing literature with good results. However, as a special class of problems in correlated alarm detection, research on first-out alarm detection is scarce.

From the ISA-18.2 standard, it is known that a first-out alarm is the first alarm that appears in the scenario of a series of alarms [25]. By analyzing the alarm sequences occurring in the alarm system, the frequent patterns of alarm sequences are mined, and the alarms appearing for the first time in these patterns are found, i.e., the first-out alarms. Showing these important patterns and the first alarms appearing in these patterns to the operator can effectively reduce the number of alarms by ignoring subsequent alarms occurring after the first-out alarms. To understand this concept better, an illustrative example is given. A sudden spike in the temperature from a boiler temperature sensor, indicating a potential issue, triggers a first alarm “High Boiler Temperature Alarm”. Then, the high boiler temperature might lead to other issues, causing a cascade of alarms. The second alarm could be “Low Water Level Alert” as the increased temperature may cause higher water evaporation. This is followed by a third alarm, “Steam Pressure Deviation”, due to the impact on steam pressure. As a result, there are multiple highly correlated alarms indicating one abnormal issue. Based on the chronological order, the alarms might unfold as Alarm 1: High Boiler Temperature Alarm, Alarm 2: Low Water Level Alert, and Alarm 3: Steam Pressure Deviation. In this scenario, “High Boiler Temperature Warning” serves as the first-out alarm, indicating the initiation of a potential problem, and the subsequent alarms provide redundant information. In real systems, such relations between the first-out alarm and subsequent alarms are not so straightforward as alarm messages are massive. Thus, detection of first-out alarms from historical alarm data is crucial for the identification of such first-out alarms.

**Figure 3:**
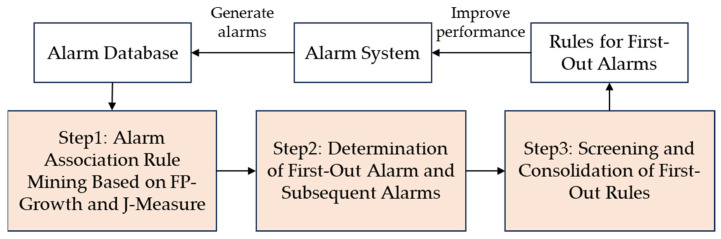
Framework of the proposed method.

In real industrial processes, first-out alarm detection can only rely on process knowledge or experienced operators; however, with the expansion of the system scale, it is difficult to judge first-out alarms by relying on manual experience. Therefore, this paper proposes to utilize association rule mining and association analysis to detect first-out alarms and export first-out rules. Figure 3 presents the framework of the proposed method. There are three key steps: First, alarm association rules are mined from the historical alarm database using FP-Growth and J-Measure. Then, first-out and subsequent alarms are determined from the association rules through a hypothesis test on the conditional probability and the consistency check of alarm occurrence orders. Last, first-out rule screening criteria are proposed to screen and consolidate first-out rules. The systematic solution for first-out alarm detection will be detailed in the next section.

## 3. Proposed Method for First-Out Alarm Detection

This section proposes the first-out alarm detection method based on the association rule mining and correlation analysis. The method consists of three major steps, namely the association rule mining, the determination of first-out subsequent alarms, and screening and consolidation of first-out rules.

### 3.1. Alarm Association Rule Mining Based on FP-Growth and J-Measure

This subsection proposes an alarm association rule mining method based on the Frequent Pattern Growth algorithm and J-Measure to detect interesting alarm association rules from the historical sequences. Some basic concepts are defined as follows: The set of unique alarms in an alarm system is denoted by $I=\left\{i_{1},i_{2},\cdots i_{n}\right\}$. An alarm set *X* contains at least one alarm in *I*, namely, X⊆I. The support degree $\theta \left(X\right)=X_{sup}$ is the percentage of historical sequences containing X to the total number of sequences in the alarm database *D*. An alarm set *X* is said to be a frequent alarm itemset if its support is no less than a given minimum support threshold s, i.e., θ(*X*)≥s. Given two alarm itemsets X,Y⊆I, the confidence *C* is calculated as the ratio of the number of sequences containing both X and Y to the number of sequences containing only *X*, i.e.,

(3) $$C\left(X\Rightarrow Y\right)=\frac{\theta (X\cup Y)}{\theta (X)}$$

where θ(X∪Y) denotes the support for co-occurrences of X,Y⊆I in the historical sequences. An alarm association rule X⇒Y is obtained if the confidence is no less than a given confidence threshold *c*, i.e., $C\left(X\Rightarrow Y\right)\geq c$.

Given an alarm database *D*, the objective of alarm association rule mining is to find strong association rules that satisfy the minimum support threshold and the minimum confidence threshold. The process includes two main steps: (1) identify all the itemsets *P* whose supports are no less than the support threshold, i.e., generating frequent itemsets; and (2) form the association rules X⇒Y based on the frequent itemsets, such that $C\left(X\Rightarrow Y\right)\geq c$; such rules are named strong association rules.

The FP-Growth algorithm is a compelling choice for mining alarm association rules from alarm and event data due to its efficiency in handling sparse datasets, memory optimization through the FP-tree structure, and scalability for large datasets [31,32]. Unlike the Apriori algorithm, FP-Growth does not rely on explicit candidate generation, contributing to its computational efficiency, particularly in scenarios where the dataset is sparse and the number of potential patterns is high, making FP-Growth well-suited for mining alarm association rules.

In addition, FP-Growth is a method designed for mining itemset patterns without considering orders, other than for sequence mining. This is because in sequence mining, the goal is to uncover sequential patterns with a focus on the order of items, which is essential for tasks involving temporal relationships. By contrast, the FP-Growth algorithm does not inherently handle temporal dependencies or ordered relationships between items. Instead, it aims at identifying frequent, unordered sets of items and does not provide mechanisms to explore patterns based on the temporal order of events. In this study, the FP-Growth algorithm is adopted to mine frequent alarm itemsets first, from which association rules can be generated by checking the confidence.

The FP-Growth algorithm does not generate candidate sets and obtains frequent itemsets through a partitioning strategy with high computational efficiency [31,32]. In FP-Growth, an FP-tree is constructed to store the frequent patterns. In the FP-tree, each node consists of a node-name, node-count, node-chain, node-link, and node-parent [31]. The pseudo code of the FP-Growth algorithm is reproduced in Algorithm 1. The procedures are explained as follows:

**Algorithm 1.** FP-Growth algorithm to produce the set of frequent alarm itemsetsInput: Alarm database *D*; Support threshold *s* Output: Set of frequent alarm itemsets *L*
Build an item header table: Scan the database *D* to obtain *L*_1_ = {*i_l_*|*θ*(*i_l_*) ≥ *s*, *l* = 1, 2, …, |*L*_1_|}, and then put *L*_1_ into an item header table and sort it in a descending order based on supports of alarms in *L*_1_.Build FP-tree: Create the root node of an FP-tree, and label it as “null”. For each transaction in *D*, the following steps are executed.Set *k* = 1 and *L* = *L*_1_.Iterative loop:      From the bottom of the item header table, sequentially find the conditional pattern base *I_k_* and its corresponding conditional pattern tree.      Mine and obtain frequent itemset *L_k_*.      Update *k* = *k* + 1, *L* = *L* ∪ *L_k_*.      Continue the loop until it is not possible to generate frequent sets or candidate sets.Return *L* = {*L_k_*: *θ*(*L_k_*) ≥ *s*, *L_k_* ⊂ *I*}.

(1) In line 1, it scans the alarm database *D* once to generate a set of frequent items and calculate their supports, sort the items in a descending order based on their supports, and generate a list of frequent items $L_{1}$.
(2) In line 2, it creates the root node of FP-tree, labeled as “null”.
(3) In lines 3–8, for each alarm sequence in the database *D*, conduct the following steps: ① Arrange the frequent items in the sequence according to the order in which they are listed, and denote the result of the arrangement as [b/B], where b is the first item and B is the list of remaining items; ② call insert_tree([b/B], *T*); ③ if B is not empty, recursively call insert_tree(*B*, *N*); procedure insert_tree([b/B], *T*) is executed as follows: if *T* has children such that *N*.node-name = *b*; then the count of *N* is increased by 1; otherwise, a new node *N* is created with its node-name set to *b*, its node-count set to 1, and its node-parent linked to its parent node *T*, and link it to a node with the same node-name through the node-chain and node-link.
(4) In line 9, all frequent itemsets $L=\{L_{k}:\theta (L_{k})\geq s,L_{k}\subset I\}$ are extracted from the alarm database *D*.

As the number of obtained itemsets is large, only those closed patterns are reserved. A frequent alarm itemset $P_{k1}$ is a closed itemset if $\theta (P_{k1})\geq s$ and there is no super pattern $P_{k2}$ such that $P_{k1}\subset P_{k2}$ with $\theta \left(P_{k2}\right)=\theta (P_{k1})$. Accordingly, for two frequent patterns $P_{k1}\in L,P_{k2}\in L$, if it is found that $\theta (P_{k1})\geq s$, $\theta \left(P_{k2}\right)=\theta (P_{k1})$, $P_{k1}\subset P_{k2}$, then $P_{k1}$ is deleted from L. For each frequent alarm itemset $P_{k}\in L$, the association rules are determined by calculating the confidences using Equation (3) and comparing them with the minimum confidence threshold, i.e., an association rule is given by

(4) $$X\Rightarrow Y\;for\;X\subset P_{k},Y\subset P_{k},X\cup Y=P_{k},if\;\theta \left(P_{k}\right)\geq s\;\&\;C\left(X\Rightarrow Y\right)\geq c$$

However, association rules satisfying the minimum support and minimum confidence thresholds are not necessarily interesting; the frequent co-occurrences of X and Y do not imply X and Y are dependent on each other, so a rule X⇒Y can be deceiving [33]. To discard such deceiving rules, a third measurement is needed to determine the interestingness of association rules, which can be achieved by correlation analysis. Since the support is essentially the probability of the occurrence of an itemset, it is straightforward to apply the information theoretic measure to assess the interestingness [34]. A basic information measure is the Shannon Entropy, which measures the relative information content of an itemset Y in the historical alarm sequences; it is defined as

(5) $$H\left(Y\right)=-p\left(Y\right)\log_{2}p\left(Y\right)-p\left(\overset{-}{Y}\right)\log_{2}p\left(\overset{-}{Y}\right)$$

where $p\left(Y\right)$ and $p\left(\overset{-}{Y}\right)$ represent the probabilities that Y is present and absent in the historical sequences, respectively. To measure the correlation between X and Y, it needs to incorporate the conditional probabilities $p\left(Y|X\right)$ and $p\left(\overset{-}{Y}|X\right)$. J-Measure as a scaled cross entropy can be used to measure the amount of information that X gives about Y, and thus is adopted here. J-Measure for X⇒ Y is defined as [35]

(6) $$J\left(X\Rightarrow Y\right)=p\left(X,Y\right)\log_{2}\frac{p\left(Y|X\right)}{p\left(Y\right)}+p\left(X,\overset{-}{Y}\right)\log_{2}\frac{p\left(\overset{-}{Y}|X\right)}{p\left(\overset{-}{Y}\right)}$$

where $p\left(X,Y\right)$ and $p\left(X,\overset{-}{Y}\right)$ denote the joint probabilities that X occurs with the presence and absence of Y in the historical sequences, respectively. The range of $J\left(X\Rightarrow Y\right)$ is [0, 1], with 0 indicating that X provides no information about Y. A higher $J\left(X\Rightarrow Y\right)$ indicates stronger correlation between X and Y for a rule X⇒Y, and thus X⇒Y is more interesting. Accordingly, those rules with $J\left(X\Rightarrow Y\right)$ equal or close to 0 should be discarded.

### 3.2. Determination of First-Out Alarm and Subsequent Alarms

From the above subsection, interesting alarm association rules are obtained from the historical sequences. Depending on the number of items, there are two types of rules: (1) The “one-to-many” rule: Given $a_{x}\in I,S\left(a_{x}\right)\subset I,a_{x}\cup S\left(a_{x}\right)=P_{k}$, there is $a_{x}\Rightarrow S(a_{x})$, such that $C\left(a_{x}\Rightarrow S(a_{x})\right)\geq c$, where $a_{x}$ denotes a single alarm and $S\left(a_{x}\right)$ denotes a subset of $P_{k}$ that excludes $a_{x}$; and (2) the “many-to-many” rule: given $A_{x}\subset I,A_{y}\subset I,A_{x}\cup A_{y}=P_{k}$, there is $A_{x}\Rightarrow A_{y}$, such that $C\left(A_{x}\Rightarrow A_{y}\right)\geq c$, where $A_{x}$ and $A_{y}$ are two different subsets of $P_{k}$. As the first-out alarm is referred to as the first occurrence of an alarm in the alarm set $S(a_{x})$, it only needs to pay attention to the strong correlation rule of multiple alarms occurring after the occurrence of a single alarm, i.e., “one-to-many” strong correlation rule, i.e., $a_{x}\Rightarrow S(a_{x})$ with $C\left(a_{x}\Rightarrow S(a_{x})\right)\geq c$. As a result, the candidate association rules for determination of first-out rules are restricted to a reasonable small number that can be well handled by the user.

Based on the “one-to-many” strong association rules obtained by association rule mining, this section determines whether each association rule is a first-out rule, which is achieved through two major steps, namely, a hypothesis test on the conditional probability for the first-out alarm and its subsequent alarms, as well as the occurrence order consistency check.

According to the ISA-18.2 standard [25], a first-out alarm is defined as the alarm that occurs for the first time in a series of alarms. Accordingly, for each alarm $a_{x}\in I$ and the set ${S(a}_{x})\subset I$, a first-out rule is formulated as

(7) $$a_{x}\Rightarrow {S(a}_{x}):\forall a_{x}\in I,{S(a}_{x})\subset I,{S(a}_{x})=\emptyset ,t(a_{x})\leq t(a_{y}),\forall a_{y}\in {S(a}_{x})$$

where $t(a_{x})$ indicates the time stamp of $a_{x}$ in a sequence of *D*. If $a_{x}$ occurs, alarms in the alarm set ${S(a}_{x})$ also occur after it; then, any alarm in the alarm set ${S(a}_{x})$ is the subsequent alarm of $a_{x}$ and alarm $a_{x}$ is the first-out alarm of ${S(a}_{x})$.

In order to determine whether $a_{x}$ is the first-out alarm of ${S(a}_{x})$, a hypothesis test is needed to determine whether $p({S(a}_{x})/a_{x})=1$ or $p(a_{x},{S(a}_{x}))=p(a_{x})$ holds. Analogous to [23,26], given $a_{x}\in I,S\left(a_{x}\right)\subset I,a_{x}\cup S\left(a_{x}\right)=P_{k}$, a hypothesis test is formulated as

(8) $$\left\{\begin{array}{c} H_{0}:p(a_{x},{S(a}_{x}))=p(a_{x})\\ H_{A}:p(a_{x},{S(a}_{x}))\neq p(a_{x}) \end{array}\right.$$

where $p(a_{x},{S(a}_{x}))$ is the joint probability that alarms $a_{x}$ and ${S(a}_{x})$ occur together as a frequent itemset $P_{k}$ in *D*; $p(a_{x})$ indicates the probability that $a_{x}$ occurs in *D*. According to [23], the log-likelihood ratio Λ can be estimated as

(9) $$\tilde{\Lambda }=\underset{N\to +\infty }{\lim}\Lambda =-2\left[k_{1}log\frac{k_{1}+k_{2}}{k_{1}}+k_{2}log\frac{k_{1}+k_{2}}{k_{2}}\right]$$

where $k_{1}$ and $k_{2}$ denote the numbers of sequences containing $a_{x}$ and $P_{k}$ in *D*, respectively. Therefore, given a threshold η, if $\tilde{\Lambda }>\eta$, then the null hypothesis $H_{0}$ does not hold, and $a_{x}\Rightarrow {S(a}_{x})$ is not a first-out rule; otherwise, if $\tilde{\Lambda }\leq \eta$, then the null hypothesis $H_{0}$ holds, and $a_{x}\Rightarrow {S(a}_{x})$ is a potential first-out rule.

Next, it should examine the order for the first-out alarm $a_{x}$ and its subsequent alarms in ${S(a}_{x})$. The frequent patterns $P_{k}$ mined by the FP-Growth algorithm are disordered, while the first-out alarm $a_{x}$ should be the earliest alarm that appears in the alarm set $P_{k}$. In order to verify whether $a_{x}\Rightarrow {S(a}_{x})$ is a true first-out rule, it is necessary to check the order in which the alarms $a_{x}$ and each alarm $a_{y}\in {S(a}_{x})$ appear in the original alarm sequences.

For any alarm sequence in the alarm database, if there exists an alarm $a_{x}$ that appears before all the alarms in the alarm set ${S(a}_{x})$, i.e., $t(a_{x})\leq t(a_{y}),\forall a_{y}\in {S(a}_{x})$, then $a_{x}\Rightarrow {S(a}_{x})$ is a true first-out rule; otherwise, $a_{x}\Rightarrow {S(a}_{x})$ is not a first-out rule. Since alarm $a_{x}$ may occur more than once in an alarm sequence, it needs to count how many times $a_{x}$ appears before all $a_{y}\in {S(a}_{x})$ in the original sequences in *D*. Suppose there are M alarm sequences in *D* and there are *m* sequences that meet $t(a_{x})\leq t(a_{y}),\forall a_{y}\in {S(a}_{x})$ for a potential first-out rule $a_{x}\Rightarrow {S(a}_{x})$. Then, the judgement condition to determine a potential first-out rule is true is formulated as

(10) $$\left\{\begin{array}{cc} a_{x}\Rightarrow {S(a}_{x})\;is\;a\;true\;rule, & if\;m/M\geq \eta_{2}\\ a_{x}\Rightarrow {S(a}_{x})\;is\;a\;false\;rule, & otherwise \end{array}\right.$$

where $\eta_{2}$ is a user-defined first-out alarm threshold; as a rule of thumb, the value of $\eta_{2}\in [0,1]$ should be closer to 1.

### 3.3. Screening and Consolidation of First-Out Rules

In Section 3.2, the first-out rules are determined from the “one-to-many” association rules that pass the hypothesis test on conditional probabilities and meet the requirement on occurrence orders of alarms in the original sequences. However, there may exist redundant rules that resemble each other. Accordingly, this subsection proposes first-out rule screening criteria to judge whether the first-out rules are redundant or not, and consolidate the results by deleting and merging redundant rules. This paper extends the two scenarios in [26] to three scenarios where first-out rules might be redundant and summarizes the first-out rule screening criteria for the corresponding scenarios.

(1) Scenario 1: Different alarms $a_{x}$ and $a_{y}$ are the first-out alarms of the same alarm set ${S(a}_{x})$, i.e.,
  (11) $$a_{x}\Rightarrow {S(a}_{x}),\;a_{y}\Rightarrow {S(a}_{x})$$
(2) Scenario 2: The first-out alarm $a_{y}$ is a subsequent alarm of another first-out alarm $a_{x}$, i.e.,
  (12) $$a_{x}\Rightarrow {S(a}_{x}),\;a_{y}\Rightarrow {S(a}_{y}),a_{y}\in {S(a}_{x})$$
(3) Scenario 3: Alarm $a_{x}$ is the first alarm of different alarm sets ${S(a}_{x})$ and ${S^{\prime }(a}_{y})$, but there exists an intersection of alarm sets ${S(a}_{x})$ and ${S^{\prime }(a}_{x})$, i.e.,
  (13) $$a_{x}\Rightarrow {S(a}_{x}),\;a_{x}\Rightarrow {S^{\prime }(a}_{x}),{S(a}_{x})\cap {S^{\prime }(a}_{x})\neq \emptyset$$

In order to reduce redundant rules, it needs to be determined whether the first-out alarm rules under all the above scenarios should be retained or merged. For these different scenarios, the first-out screening criteria are summarized as follows:

(1) In Scenario 1, it is known that $a_{x}\Rightarrow {S(a}_{x})$, $a_{y}\Rightarrow {S(a}_{y})$, $a_{x}\notin {S(a}_{y})$, $a_{y}\notin {S(a}_{x})$, ${S(a}_{x})={S(a}_{y})$. Thus, for $a_{z}\in {S(a}_{x})$, $p(a_{x}/a_{y})\neq 1,$ $p(a_{y}/a_{x})\neq 1,$ $p(a_{z}/a_{y})=1,$ and $p(a_{z}/a_{x})=1$. If $a_{x}\Rightarrow a_{z}$ implies $a_{y}\Rightarrow a_{z}$ and $a_{y}\Rightarrow a_{z}$ implies $a_{x}\Rightarrow a_{z}$, then $a_{y}\Rightarrow a_{z}$ and $a_{x}\Rightarrow a_{z}$ are redundant, and only one rule should be retained. Otherwise, $a_{y}\Rightarrow a_{z}$ and $a_{x}\Rightarrow a_{z}$ are distinct rules, and should both be preserved. In this scenario, it should be checked whether $a_{x}$ and $a_{y}$ are redundant or not, which can be achieved through hypothesis test on $p(a_{x},a_{y})=p(a_{x})$.
(2) In Scenario 2, it is known that $a_{x}\Rightarrow {S(a}_{x})$, $a_{y}\Rightarrow {S(a}_{y}),a_{y}\in {S(a}_{x})$. Then, ${S(a}_{x})$ can be extended to ${\tilde{S}(a}_{x})={S(a}_{x})\cup {S(a}_{y})$, and thus we can obtain $a_{x}\Rightarrow {\tilde{S}(a}_{x})$, and it needs to be determined whether to retain $a_{y}\Rightarrow {S(a}_{y})$. If $a_{y}$ always occurs after $a_{x}$, then one of the rules is duplicated and $a_{y}\Rightarrow {S(a}_{y})$ should be deleted. Otherwise, $a_{y}\Rightarrow {S(a}_{y})$ should be retained. In this scenario, it should be checked whether $a_{x}$ and $a_{y}$ hold a strong causal relation; as $a_{y}$ must follow $a_{x}$ as reflected by $a_{x}\Rightarrow {S(a}_{x})$, $a_{y}\in {S(a}_{x})$, it is still necessary to check whether $a_{x}$ and $a_{y}$ are redundant.
(3) In Scenario 3, it is known that $a_{x}\Rightarrow {S(a}_{x})$, $a_{x}\Rightarrow {S^{\prime }(a}_{x})$, ${S(a}_{x})\cap {S^{\prime }(a}_{x})\neq \emptyset$. Let ${{\tilde{S}(a}_{x})=S(a}_{x})\cup {S^{\prime }(a}_{x})$; then it needs to be determined whether $a_{x}\Rightarrow {\tilde{S}(a}_{x})$ is valid. If $a_{x}\Rightarrow {S(a}_{x})$ occurs and also $a_{x}\Rightarrow {S^{\prime }(a}_{x})$ occurs, then the two first-out rules can be combined, i.e., $a_{x}\Rightarrow {\tilde{S}(a}_{x})$. Otherwise, both $a_{x}\Rightarrow {S(a}_{x})$ and $a_{x}\Rightarrow {S(a}_{y})$ should be preserved. In this scenario, it should be checked whether all alarms in ${S(a}_{x})\cup {S^{\prime }(a}_{x})$ always appear together in historical sequences.

As a result, consolidated first-out rules are screened out from the initial rules by deleting or merging redundant rules. Then, the user can focus on a smaller number of more meaningful first-out rules, which can help the user to recognize the most important alarms in a series of alarms and ignore invalid or redundant alarm notifications.

### 3.4. Discussions

This subsection mainly introduces the differences between the proposed method and the existing alarm correlation analysis methods, discusses the scalability of the proposed method, and then presents the challenges faced during the testing phase.

Even though the proposed method exploits correlation analysis for the detection of first-out alarms, it is very different from the existing alarm correlation analysis methods as the objectives and exploited strategies are disparate. To justify the novelty and superiority of the proposed method, Table 2 lists and compares it with existing approaches to alarm correlation analysis. The differences lie in the following aspects:

(1) The exploited data types are different. The data for alarm correlation analysis are binary alarm time series over a certain consecutive period. Regarding first-out alarm detection, the required data are essentially a collection of alarm sequences.
(2) The objectives are different. The proposed method aims at identifying first-out alarms and exporting first-out rules, whereas existing alarm correlation analysis methods measure and export the correlations between alarms.
(3) The principles are different. The detection of first-out alarms usually involves multiple alarms and requires alarm order information, while existing alarm correlation analysis methods only explore the correlation between two alarms and do not consider orders between alarm occurrences.

It can be seen that even though first-out rules imply alarm correlations, there are obvious differences between the detection methods. Well-formulated existing alarm correlation analysis cannot be applied for identification of first-out alarms. Thus, the proposed method is specially designed and exhibits significant novelties in detecting first-out alarms.

Further, in real-world applications, the testing phase for the proposed method for detecting first-out alarms may encounter some limitations and challenges that could impact the accuracy and reliability of the results. Here, three challenges are summarized:

(1) Time stamps of alarm events are key information. Inaccurate or inconsistent time stamps can lead to misinterpretations of the temporal order of alarms.
(2) Noisy or incomplete historical data may hinder the ability to accurately assess and validate the first-out alarm detection method.
(3) Transitioning the method from a testing environment to a real-time implementation may pose scalability challenges.

Thus, addressing these challenges during the testing phase is essential for refining and improving the robustness of the first-out alarm detection method, ensuring its effectiveness in diverse industrial environments. In view of the first two challenges, the foremost step is to improve the data quality, which can be achieved by reducing chattering alarms and removing incomplete messages in the historical alarm data. Furthermore, the scalability challenges can be handled according to the following discussion.

When implementing the proposed method for detection of first-out alarms, scalability must be considered as the volume of alarm data can be massive in real-world industrial systems. Thus, for application to large-scale systems, the following guidelines in terms of computational resources and runtime can be considered:

(1) The data retrieval process can be optimized by using a database indexing system and leveraging efficient data structures such as hash tables or tree structures, to expedite the search for the earliest occurrences of each alarm.
(2) Data pruning or filtering can be applied first to preprocess the alarm data and eliminate redundant information; thus the computational burden in the subsequent analysis can be reduced.
(3) The detection task can be portioned into sub-tasks based on the units or groups that alarms belong to, and thus the proposed method can work efficiently for each sub-task.

## 4. Case Study

In this section, alarm data obtained from a public industrial simulation model are used to validate the effectiveness of the proposed first-out alarm detection method.

### 4.1. Experiment Preparation

The Vinyl Acetate Monomer (VAM) industrial simulation model is based on a typical full-flow open-source chemical platform, which has a full set of standard units required for the production of VAM [36,37]. The model can simulate the actual chemical production, providing users with a realistic operating environment. Both steady and unsteady states are available in the simulation model, which is implemented through a visual modeler that allows the introduction of anomalies in steady-state operation, a real time window monitor that allows the user to monitor the process and equipment operating status, and a data interface to extract data for analysis and secondary processing. The industrial simulation model of “Visual Modeler” is available through Omega Simulation Co., Ltd.’s website at www.omegasim.co.jp (accessed on 15 December 2023). The user can activate single/multiple faults manually or automatically at any time and adjust the impact of disturbances or faults by setting status parameters including variable percentage, time constant, and fault mode.

**Figure 4:**
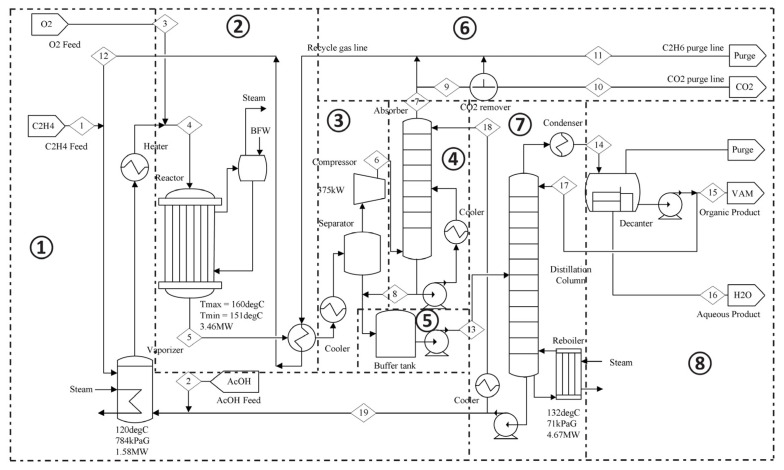
Process flow diagram of the VAM industrial simulation model, which has eight sections separated by dashed lines.

The process flow diagram of the VAM simulation model is shown in Figure 4. It consists of eight sections, namely the Raw Material Feed, Reactor, Separator and Compressor, Absorber, Buffer Tank, CO_2_ Remover and Purge Line, Distillation Column, and Decanter [36,37,38]. The Raw Material Feed section introduces raw materials into the Reactor, where they undergo mixing to produce the VAM product. Then, the Separator divides the gas exiting the reactor into the VAM crude and recycle gas; the latter is compressed and circulated by the Compressor. Next, the Absorber absorbs the uncompressed VAM from the recycle gas and feeds the compressed crude to the Buffer Tank, which then feeds the crude to the Distillation Column. The CO_2_ Remover and Purge Line eliminates Carbon Dioxide, a by-product, and replenishes Ethane from the recycle gas to the raw material feed. The Distillation Column separates the crude into a VAM–Water mixture and acetic acid. Last, the Decanter separates the liquid VAM and water.

To extract alarm data, a simulated alarm system is configured by setting alarm thresholds for the monitored process variables based on the setting in [38,39]. The model has 14 types of disturbances and 22 types of faults. In the data acquisition, the configuration time of the faults was set to vary from 20 to 60 min. Then, 130 alarm sequences were extracted by triggering the faults; among the 22 fault types, 13 faults were associated with significant long sequences and thus were used. Details about generating alarm data can be found in [38,39]. Given the collected data generated from the VAM, the proposed first-out alarm detection method was exploited to extract the frequent patterns in the alarm sequences, keep the interesting association rules, and obtain the first-out rules. The specific experimental steps are as follows:

(1) The method in Section 3.1 was applied to extract alarm association rules. The minimum support degree and the minimum confidence level were set to 0.95 and 0.99, respectively. Initially, a total of 673,588 frequent patterns could be obtained. By keeping only closed alarm patterns and then identifying interesting association rules, 3104 rules were reserved from the historical alarm sequences.
(2) The method in Section 3.2 was exploited to determine first-out rules. In the hypothesis test, the significance level was set to 0.05, and the corresponding $\chi^{2}$ threshold *η* was 3.84. The satisfaction rate threshold $\eta_{2}$ was set as 0.9. As a result, 1746 first-out rules were identified based on the interesting alarm association rules.
(3) The method in Section 3.3 was utilized to screen and consolidate first-out rules, so as to reduce the redundancy in the results. Eventually, 204 consolidated first-out rules were received.

**Figure 5:**
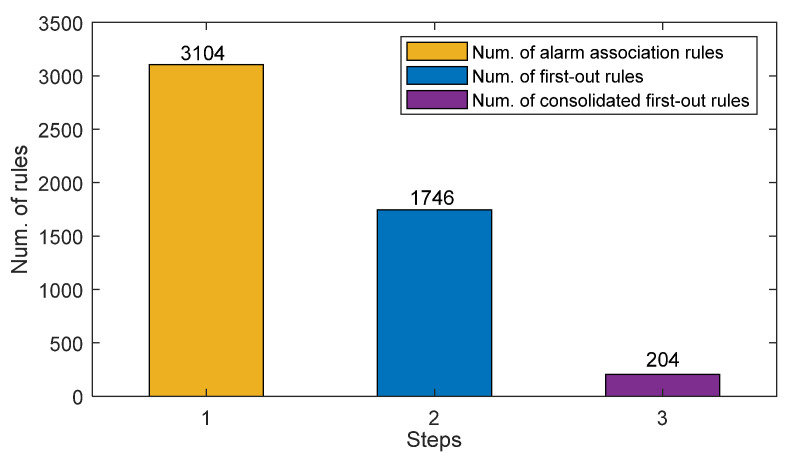
Numbers of extracted alarm association rules, first-out rules, and consolidated first-out rules according to the steps in Section 3.1, Section 3.2, and Section 3.3, respectively.

Figure 5 presents the numbers of extracted alarm association rules, first-out rules, and consolidated first-out rules according to the steps in Section 3.1, Section 3.2, and Section 3.3, respectively. It can be seen that the reduction in rules is significant, making the first-out rules in the final results less redundant. It should be noticed that validating the results is a common and difficult problem in data mining [21,22,23,24]. It usually exploits standard metrics such as support and confidence to evaluate whether the data mining is effective. In this case study, all the extracted first-out rules satisfy both the minimum thresholds, implying that the results are significant and reasonable. However, verifying the correctness of the extracted rules is hard because the exploited data are unlabeled and sufficient knowledge is required for verification. Accordingly, some examples of first-out rule screening under different scenarios are presented in the following subsections, and the correctness of the results is verified by process knowledge of the alarms.

### 4.2. Scenario 1

**Figure 6:**
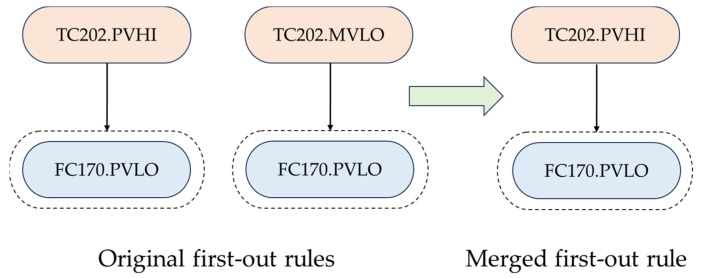
An example of first-out rules that should be merged under Scenario 1.

**Figure 7:**
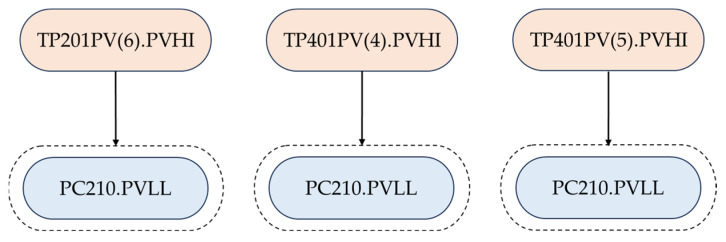
An example of first-out rules that should be retained under Scenario 1.

In Scenario 1, different alarms $a_{x}$ and $a_{y}$ are the first-out alarms of the same alarm set ${S(a}_{z})$; it then needs to be determined whether $a_{x}\Rightarrow {S(a}_{z})$ and $a_{y}\Rightarrow {S(a}_{z})$ are redundant. Two different results are presented. In Figure 6, alarms TC202.PVHI and TC202.MVLO are the first-out alarms for FC170.PVLO, but they hold a redundancy relationship. Thus, only one of them is kept by merging the two rules into one. The same conclusion is also drawn for first-out rules TP201PV(6).PVHI ⇒ QI400.PVHI and QI204.PVHI ⇒ QI400.PVHI, as they hold significant redundancy according to the hypothesis test. In Figure 7, TP201PV(6).PVHI, TP401PV(4).PVHI, and TP401PV(5).PVHI are the first-out alarms for PC210.PVLL, and after hypothesis testing, it is found that there is no redundancy relationship among the three, so that all the three rules need to be retained.

To validate the result, it needs to be determined how these alarms are generated to form such relations. Regarding the result in Figure 6, TC202.PVHI and TC202.MVLO indicate that the control variable and the manipulating variable of the same process tag (namely, the temperature of the reactor’s catalyst bed) increase and exceed the alarm thresholds, triggering the two alarms. The two alarms are related to the same tag and always occur almost simultaneously. Further, the alarm FC170.PVLO denotes that the oxygen feed flow rate decreases when the temperature of the reactor’s catalyst bed becomes abnormal, and accordingly, FC170.PVLO happens after TC202.PVHI and TC202.MVLO. Thus, the result in Figure 6 is reasonable.

In Figure 7, TP201PV(6).PVHI, TP401PV(4).PVHI, and TP401PV(5).PVHI denote that the temperatures in the reactor (sensor 6), absorber (sensor 4), and absorber (sensor 5) reach a high level and exceed the high alarm limits, thus triggering the corresponding alarms. The alarm TP201PV(6).PVHI is located at a different unit compared to TP401PV(4).PVHI and TP401PV(5).PVHI, and thus their occurrences are not correlated. Alarms TP401PV(4).PVHI and TP401PV(5).PVHI are located at different positions of the reactor and thus also have low correlations. Whenever one of the above temperatures reaches a high level, the pressure controller PC210 in the reactor responds to export low values and triggers the extremely low alarm PC210.PVLL. Thus, the result in Figure 7 is reasonable.

### 4.3. Scenario 2

**Figure 8:**
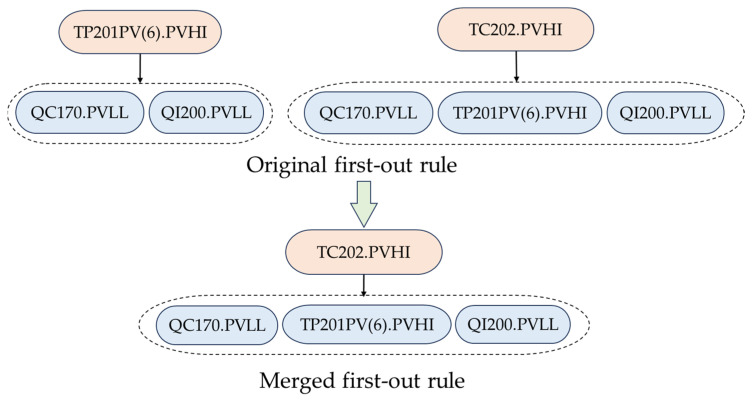
An example of first-out rules that should be merged under Scenario 2.

In Scenario 2, the first-out alarm $a_{y}$ is the consequential alarm of another first-out alarm $a_{x}$, i.e., $a_{x}\Rightarrow {S(a}_{x}),a_{y}\Rightarrow {S(a}_{y})$, $a_{y}\in {S(a}_{x})$. Under this scenario, an example of first-out rules that should be merged is presented. In Figure 8, TP201PV(6).PVHI is the first-out alarm for QC170.PVLL and QI200.PVLL, and TC202.PVHI is the first-out alarm for TP201PV(6).PVHI, QC170.PVLL and QI200.PVLL. Thus, it needs to be determined whether these two first-out rules should be merged into one first-out rule. According to the hypothesis test, it is found that these two first-out rules hold a significant redundancy relationship and thus should be merged into one first-out rule, i.e., TC202.PVHI ⇒ {TP201PV(6).PVHI, QC170.PVLL, QI200.PVLL}.

In this scenario, QC170 denotes the oxygen density of the raw material feed with the fresh oxygen feed, TP201PV(6) indicates the temperature in the reactor, and QI200 represents the oxygen concentration from the reactor outlet gas to the separator. When the controlled temperature of the reactor’s catalyst bed reaches a high level and goes beyond the high alarm limit, the alarm TC202.PVHI is triggered. As a result, the temperature in the reactor increases to trigger TP201PV(6).PVHI; by contrast, QC170 and QI200 decrease drastically to trigger QC170.PVLL and QI200.PVLL, respectively. Thus, the result in Figure 8 is reasonable.

### 4.4. Scenario 3

**Figure 9:**
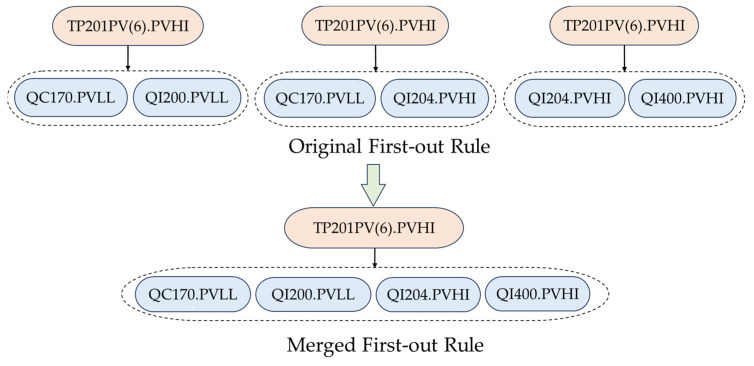
An example of first-out rules that should be merged under Scenario 3.

In Scenario 3, alarm $a_{x}$ is the first-out alarm of different alarm sets ${S(a}_{x})$ and ${S^{\prime }(a}_{x})$, but there exists an intersection of alarm sets ${S(a}_{x})$ and ${S(a}_{y})$, i.e., $a_{x}\Rightarrow {S(a}_{x})$, $a_{x}\Rightarrow {S^{\prime }(a}_{x})$, and ${S(a}_{x})\cap {S^{\prime }(a}_{x})\neq \emptyset$. Then, it needs to be judged whether $a_{x}\Rightarrow {S(a}_{x})$ and $a_{x}\Rightarrow {S^{\prime }(a}_{x})$ are redundant. From the studied dataset, two different results were obtained under Scenario 3. In Figure 9, TP201PV(6).PVHI is the first-out alarm for QC170.PVLL and QI200.PVLL, the first-out alarm for QC170.PVLL and QI204.PVHI, and also the first-out alarm for QI204.PVHI and QI400.PVHI. It has been validated that all these subsequent alarms appear together in historical sequences with a high probability, and thus they are merged into one rule TP201PV(6).PVHI ⇒{QC170.PVLL, QI200.PVLL, QI204.PVHI, QI400.PVHI}.

In the scenario of Figure 9, when the temperature of the reactor reaches a high level and triggers the alarm TP201PV(6).PVHI, four quality- or composition-related alarms (QC170.PVLL, QI200.PVLL, QI204.PVLL, and QI400.PVLL) are very likely to occur afterward. Here, QI204 denotes the AcOH concentration from the reactor outlet gas to the separator and QI400 indicates the oxygen concentration from the recycle gas to the reactor outlet gas cooler. These process tags are highly associated with respect to the material flow and are related to the temperature abnormality in the reactor. Thus, the obtained result in Figure 9 is reasonable.

**Figure 10:**
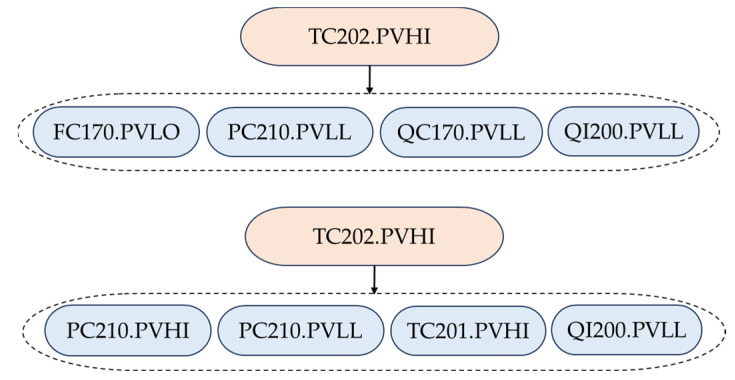
An example of first-out rules that should be retained under Scenario 3.

In Figure 10, TC202.PVHI is the first-out alarm for FC170.PVLO, PC210.PVLL, QC170.PVLL, and QI200.PVLL. It is also the first-out alarm for PC210.PVHI, PC210.PVLL, TC201.PVHI, and QI200.PVLL. It has been validated that these subsequent alarms appear together in historical sequences with a low probability, and thus both of the first-out rules should be retained.

The obtained result in this scenario is reasonable because of the following reason. There are various causes of the high control temperature of the reactor’s catalyst bed. In different occasions, the severities could be different. As a result, different subsequent alarms may appear. In the upper subplot, the controlled oxygen feed flow rate FC170 and the oxygen density QC170 reach a low level due to the reaction of the closed loop control. In the lower subplot, the controller pressure and temperature in the reactor increase due to a different control strategy. It should also be noted that PC210 and QI200 are highly correlated with TC202, and thus PC210.PVLL and QI200.PVLL always appear after TC202.PVHI.

## 5. Conclusions

This paper proposes a first-out alarm detection method based on association rule mining and correlation analysis. First, an alarm association rule mining method based on FP-Growth and J-Measure is proposed to extract interesting alarm association rules from historical sequences. Then, the first-out alarms and subsequent alarms are determined through correlation analysis in the form of a hypothesis test on the conditional probability for alarm occurrences. Last, criteria for screening and consolidation of first-out rules are presented to merge redundant first-out rules and delete invalid rules. The proposed method was tested via a case study with data obtained from a public chemical simulation plant. According to the results, the proposed method successfully detected the first-out rules based on the strong association rules mined from the historical alarm sequences. Such results would provide decision-making support for the design and operation of the alarm system, so as to reduce nuisance alarms and alleviate alarm overloading problem.

There exist some problems in this research that deserve future exploration: (1) Validating results is a common and difficult issue for data mining methods, and developing an effective way to measure the accuracy of the first-out rules in this work rather than relying on process knowledge is a hard problem that should be explored. (2) Future work could focus on exploring the scalability of the proposed first-out alarm detection method for large-scale industrial alarm datasets. (3) Integrating insights from domain experts and conducting case studies in real-world industrial settings could provide valuable context-specific refinements for broader utility.

## Figures and Tables

**Table 1:** An example of historical alarm and event data.

No.	Alarm Tags	Time Stamps	Priority	Units
1	TC202.MVLO	22 April 2022 10:37:04	High	Reactor
2	TC202.SVHH	22 April 2022 10:37:38	Low	Reactor
3	TC150.MVLO	22 April 2022 10:39:20	High	Feedstock
4	TC150.MVLL	22 April 2022 10:40:11	Low	Feedstock
5	TP401PV(6).PVLO	22 April 2022 10:40:34	Low	Feedstock
6	TP401PV(6).PVHI	22 April 2022 10:51:22	Critical	Feedstock
7	TC410.PVHI	22 April 2022 10:55:21	Critical	Reactor
8	TP401PV(6).PVLO	22 April 2022 11:03:34	Low	Reactor
9	FC420.MVHH	22 April 2022 11:06:31	Low	Reactor

**Table 2:** Comparison between the proposed first-out alarm detection method and the existing alarm correlation analysis methods.

Method	Objective	Main Algorithms and Strategies	Type of Data Inputs	Detect Relations in Pair or Not	Consider Orders or Not
Proposed first-out alarm detection method	Identify first-out alarms and export first-out rules	Association rule mining, J-Measure, hypothesis test, and screening criteria	Sequences of alarm events	No	Yes
Alarm correlation analysis in [4,5]	Detect correlated alarms and calculate similarity coefficients	Cross-correlation function, Sorgenfrei and Jaccard coefficients	Binary valued alarm signals	Yes	No
Alarm correlation analysis in [7,8]	Detect correlated alarms and measure correlation levels	Gaussian kernel function, Pearson’s correlation coefficient, and estimation of correlation delay	Continuous valued pseudo alarm signals	Yes	No
Alarm correlation analysis in [9,10]	Detect correlated alarms and measure correlation levels	Cross-correlation function, partition of time sequences, matching of sequence blocks	Time-stamped alarm signals	Yes	No
Alarm correlation analysis in [11]	Detect correlated alarms and measure correlation levels	Calculation of conditional probabilities	Multi-alarm-state sequences	Yes	No

## Data Availability

No new data were created or analyzed in this study. Data sharing is not applicable to this article.
